# Age- and region-specific gut microbiota dysbiosis in axial spondyloarthritis: a systematic review and meta-analysis

**DOI:** 10.3389/fimmu.2026.1736358

**Published:** 2026-03-16

**Authors:** Lidi Liu, Liquan Yin, Zhicheng Liu, Mengmeng Xu, Fanshi Kong, Yinhua Zheng

**Affiliations:** 1 Department of Spinal Surgery, The First Hospital of Jilin University, Changchun, China; 2 Department of Rehabilitation Medicine, China-Japan Union Hospital of Jilin University, Changchun, China; 3 General Surgery Center, The First Hospital of Jilin University, Changchun, China; 4 Department of Rehabilitation Medicine, The First Hospital of Jilin University, Changchun, China

**Keywords:** ankylosing spondylitis, axial spondyloarthritis, gut microbiota, inflammation, meta-analysis

## Abstract

**Background:**

Axial spondyloarthritis (axSpA) is a chronic inflammatory disease affecting the spine and sacroiliac joints. Emerging evidence suggests that the gut microbiota may contribute to its pathogenesis, but findings on microbial diversity and composition remain inconsistent.

**Methods:**

We systematically searched PubMed, Embase, Web of Science, and the Cochrane Library to identify studies comparing the gut microbiota between adults with axSpA and healthy controls. α-Diversity (Observed richness, Shannon, Simpson, Chao1, ACE) and microbial composition at the phylum level were extracted. Random-effects meta-analyses were performed using standardized mean differences (SMDs), with subgroup analyses and robustness checks (prespecified sensitivity and leave-one-out analyses).

**Results:**

Twenty-five studies (n = 1639) were included. Compared with controls, axSpA was associated with lower α-diversity for Observed richness (SMD = −0.59, *P* = 0.04), Shannon (SMD = −0.19, *P* = 0.03), and Chao1 (SMD = −0.51, *P* = 0.02), while Simpson (SMD = −0.23, *P* = 0.12) and ACE (SMD = 0.42, *P* = 0.14) showed no significant differences. In age-stratified analyses, reductions were more apparent in participants >40 years (Shannon: SMD = −0.38, *P* = 0.002; Simpson: SMD = −0.50, *P* = 0.001). Sensitivity and leave-one-out analyses were directionally consistent; however, observed richness and Shannon became non-significant after excluding non-comparable datasets. Phylum-level findings were summarized descriptively, suggesting lower Bacteroidota and Actinobacteriota and higher Proteobacteria in axSpA.

**Conclusion:**

axSpA is associated with reduced α-diversity and compositional shifts in gut microbiota, but the magnitude of effects varies across studies. Heterogeneity and methodological differences warrant cautious interpretation. Longitudinal and functional studies are needed to clarify causality and therapeutic implications.

**Systematic Review Registration:**

https://www.crd.york.ac.uk/prospero/, identifier CRD420251089098.

## Introduction

Axial spondyloarthritis (axSpA) is a chronic, immune-mediated inflammatory disorder that primarily affects the sacroiliac joints and spine. It includes both non-radiographic axSpA (nr-axSpA) and radiographic form, commonly referred to as ankylosing spondylitis (AS) (1). The pathogenesis of axSpA is multifactorial. Genetic susceptibility, most prominently HLA-B27, plays a pivotal role (2). At the same time, the incomplete penetrance of HLA-B27 suggests the importance of environmental influences in disease initiation and progression (3).

Among these environmental factors, the gut microbiome has attracted growing attention. This diverse microbial community interacts closely with the host, and it has been increasingly implicated in axSpA pathophysiology (4, 5). The clinical overlap between axSpA and inflammatory bowel disease, together with evidence of subclinical gut inflammation in many patients, lends support to the concept of the gut–joint axis (6, 7). Advances in high-throughput sequencing have further revealed that patients with axSpA exhibit distinct gut microbial profiles versus healthy controls (8, 9). Typically, these changes include a reduction in overall microbial diversity, which is often considered a marker of ecological imbalance (10), together with specific compositional alterations, including the depletion of butyrate-producing taxa (e.g., *Faecalibacterium prausnitzii)* and enrichment of pro-inflammatory pathobionts (11).

The mechanisms linking microbial imbalance and joint inflammation are still being clarified. Dysbiosis can compromise intestinal barrier integrity, facilitate microbial translocation, and activate mucosal immune responses, especially through the IL-23/IL-17 pathway (12, 13). Microbial metabolites, particularly short-chain fatty acids (SCFAs), are also important as they influence immune regulation, inflammatory tone, and bone homeostasis (14, 15).

Despite numerous case–control studies and several meta-analyses, findings on α-diversity (*e.g.*, Shannon index) and taxonomic shifts (*e.g.*, Bacteroidota abundance) remain inconsistent. These discrepancies are likely attributable to unexplored heterogeneity in patient characteristics, including age, as older patients may present with longer disease durations and experience greater exposure to medications; geographic region, as Western and Asian populations differ substantially concerning dietary patterns, particularly fiber and processed food intake; and disease phenotype, as nr-axSpA and AS may reflect different stages or severities of inflammation (16, 60). Because gut microbial ecology is strongly shaped by age and geography, we prespecified age- and region-stratified subgroup analyses to explore whether disease-associated signals are context-dependent across cohorts. Previous meta-analyses have not systematically addressed these factors, thereby restricting a comprehensive understanding of the gut–joint axis in axSpA.

To address this knowledge gap, we performed a systematic review and meta-analysis to quantify alterations in the gut microbiota of patients with axSpA and evaluate the influence of age, geographic regions, and disease phenotypes on these changes. To our knowledge, this represents the first meta-analysis to systematically examine these factors, providing novel insights into the contribution of the gut microbiota to axSpA pathogenesis.

## Methods

This meta-analysis followed the recommendations of the Cochrane Handbook for Systematic Reviews of Interventions and the PRISMA statement (17, 18). The study protocol was prospectively registered in PROSPERO (CRD420251089098).

### Literature search

We conducted a systematic search of PubMed, Embase, Web of Science, and the Cochrane Library from inception to August 19, 2025. The search combined two groups of keywords: (1) “Axial Spondyloarthritis” OR “Ankylosing Spondylitis” OR “Spondylitis, Ankylosing” and (2) “Gastrointestinal Microbiome” OR “Gut Microflora” OR “Gastrointestinal Microflora” OR “Intestinal Microbiome.” Only human studies published as full-length articles in English peer-reviewed journals were considered. The reference lists of relevant original and review articles were also screened to capture additional eligible studies. The detailed strategy is provided in **Supplementary Table 1**.

### Inclusion criteria

Eligible studies were required to meet the following criteria: investigated gut microbiota alterations in patients with axSpA/AS; included adults diagnosed using recognized clinical criteria (*e.g.*, modified New York, ASAS) and a healthy control group; defined healthy controls as individuals with no history of inflammatory bowel disease, autoimmune disorders, or chronic infections; no recent (within 3 months) use of antibiotics, probiotics, or immunosuppressants known to influence gut microbiota; and self-reported normal bowel habits (≤ 3 bowel movements/day, without diarrhea or constipation); reported at least one relevant outcome (α-diversity indices, relative abundance of taxa, or methodological details of fecal sample handling); used 16S rRNA gene sequencing and/or whole-metagenome shotgun sequencing to characterize the gut microbiota; employed an observational design (cross-sectional, case–control, or cohort); and published as full-length, peer-reviewed journal articles in English.

### Exclusion criteria

Studies were excluded if they met any of the following criteria: duplicate reports; studies involving participants <18 years old; studies without a healthy control group; studies failing to report α-diversity or compositional data; studies lacking sufficient data or unavailable in full text; and reviews, editorials, preclinical studies, or conference abstracts.

### Study selection and data extraction

Two reviewers independently screened titles/abstracts and extracted data using a standardized form. Disagreements were resolved by consensus or consultation with a third investigator. The extracted information included first author, year of publication, country/region, sample size, demographic characteristics, diagnostic criteria, treatments, sequencing platform/region, α-diversity outcomes and taxonomic composition at the phylum level. When methods were unclear, attempts were made to contact the study authors for clarification.

### Quality assessment

Methodological quality was evaluated using the Newcastle–Ottawa Scale (NOS) (19, 20), which considers selection, comparability, and outcome/exposure. Studies scoring >6 (maximum, 9) were considered high quality. Two reviewers performed the assessment independently, and discrepancies were resolved through discussion or a third reviewer.

### Statistical analysis

Meta-analyses were conducted following the recommendations of the Cochrane Handbook for Systematic Reviews of Interventions (21). Pooled estimates were expressed as standardized mean differences (SMDs) with 95% confidence intervals (CIs). Statistical heterogeneity was assessed using the *I^2^* statistic and interpreted according to Cochrane Handbook guidelines for non-randomized studies as follows: low, 0%–40%; moderate, 30%–60%; substantial, 50%–90%; and considerable, 75%–100%. Given the inherent methodological and clinical variability in observational studies, including differences in study populations, sequencing methods, and sample processing, random-effects models were applied to all analyses irrespective of the level of heterogeneity. All statistical analyses were conducted in RevMan (version 5.4, Cochrane Collaboration, Oxford, UK). *P* < 0.05 was considered statistically significant.

## Results

### Basic characteristics and quality assessment

**Figure 1:**
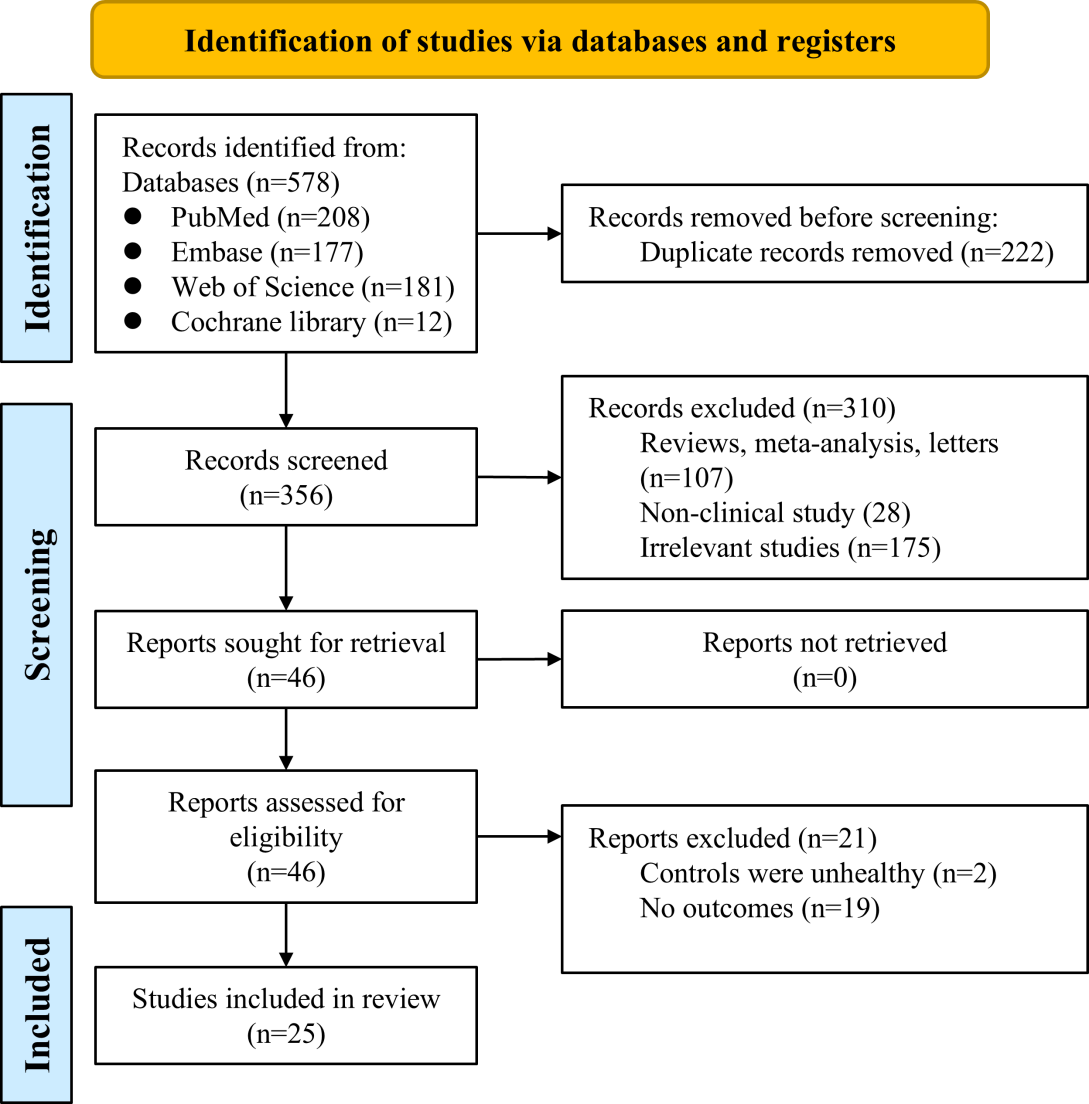
PRISMA flowchart presenting the study selection process for this systematic review and meta-analysis.

The PRISMA flowchart of the study selection process is summarized in **Figure 1**. The initial search identified 578 records from PubMed, Embase, Web of Science, and the Cochrane Library. After excluding 222 duplicates and 107 reviews, meta-analyses, and letters, 249 articles remained. Of these, 175 were excluded after screening the titles and abstracts, and 28 non-clinical studies were further excluded. Ultimately, 46 full texts were reviewed in detail, and 21 were further excluded, leaving 25 eligible studies for quantitative synthesis (60, 18–22).

The included studies were published between 2015 and 2025. The majority of studies were conducted in China (n = 15), followed by the USA (n = 2), France (n = 2), Australia, Belgium, Colombia, Korea, Romania, and the UK (n = 1 each). Collectively, these studies involved 1639 participants. Patients were diagnosed using recognized criteria such as the ASAS classification criteria, the modified New York criteria, and national guidelines. Commonly reported treatments included nonsteroidal anti-inflammatory drugs (NSAIDs), biologics (anti-TNF agents), and disease-modifying antirheumatic drugs.

Gut microbiome profiling was mainly performed using 16S rRNA sequencing and/or whole-metagenome shotgun sequencing, most often targeting the V3–V4 or V4 regions on Illumina platforms. Taxonomic assignment relied on reference databases such as SILVA, Greengenes, and RDP. α-Diversity was assessed using the Shannon, Simpson, Chao1, and Observed richness indices, and taxonomic composition (phylum level). All studies scored 7–8 on the NOS, suggesting a generally low risk of bias (**Tables 1**, **2**).

**Table 1:** Characteristics of the included studies.

Study	Country	Design	Sample size	Age (years)	Male (%)	Diagnostic standard	Concomitant treatment	Sequencing platform	Sequencing region	Database used	α-Diversity index
Berland 2023	France	CC	axSpA: 102; HC: 63	50 ± 11.3	49.0	ASAS criteria	NSAIDs: 53.5%, glucocorticoids: 5.0%, DMARDs: 4.0%, anti-TNF: 28.4%, antacids: 32.7%	5500 SOLiD Wildfire	Whole-metagenome shotgun	IGC2 gene catalog, KEGG, eggNOG, TIGRFAMs	Observed species, Shannon
Breban 2017	France	CC	axSpA: 49; HC: 18	48 ± 13	45.0	ASAS criteria	NSAIDs: 51%; corticosteroids: 12.2%; DMARDs: 4%; biotherapy: 30.6%; antiacids: 30.6%	Roche GS-FLX 454 pyrosequencer	V3–V4 regions of the 16S rRNA gene	Greengenes	Observed species, Shannon, Simpson, Chao1
Chen 2019	China	CC	AS: 41; HC: 19	28.94 ± 10.29	85.4	1984 modified New York criteria	NR	Illumina HiSeq 2500	V4 region of the 16S rRNA gene	SILVA	Shannon, Simpson
Chen 2021	China	PC	AS: 30; HC: 24	31.23 ± 7.48	90.0	1984 modified New York criteria	NSAIDs: 66.7%; sulfasalazine: 16.7%	Illumina HiSeq 2500	V4 region of the 16S rRNA gene	SILVA	Shannon, Simpson
Costello 2015	Australia	CC	AS: 9; HC: 9	33.0 ± 9.1	78	Modified New York criteria	One patient with AS on NSAIDs; three patients with occasional/interrupted NSAID use; remaining patients on paracetamol ± tramadol	Illumina MiSeq	V4 region of the 16S rRNA gene	Greengenes	Observed species
Dai 2022	China	PC	AS: 24; HC: 11	32.3 ± 10.5	91.7	Clinical and imaging criteria	Anti-TNF-α agents	Illumina HiSeq X10	V3–V4 regions of the 16S rRNA gene	SILVA, Greengenes	Simpson index
Gill 2022	USA	PC	axSpA: 17; HC: 14	58	59.0	Modified New York criteria and ASAS criteria	Biologics, NSAIDs	Illumina MiSeq	V4 region of the 16S rRNA gene	SILVA	Observed, Shannon
Li 2019	China	CC	AS: 22; HC: 19	34.86 ± 10.75	100.0	New York criteria	NSAIDs: 40.9%; etanercept: 36.4%, treatment-naïve: 22.7%	Illumina HiSeq PE250	V3–V4 regions of the 16S rRNA gene	RDP Classifier 2.8	Observed species, Shannon, Simpson
Li 2023	China	CC	AS: 113; HC: 37	NR	NR	NR	NR	Metagenomic shotgun sequencing	Whole-metagenome shotgun	Chinese gut virus catalog, KEGG	Shannon, Simpson
Liu 2020	China	CC	AS: 10; HC: 12	49.5 ± 6.2	60.0	Modified New York criteria	No NSAIDs, acetaminophen, or tramadol	Illumina HiSeq 2500	V3–V4 regions of the 16S rRNA gene	Greengenes	Shannon, Simpson, Chao1, ACE
Marquez-Ortiz 2023	Colombia	CC	axSpA: 32; HC: 7	33.2	56.2	ASAS criteria and ESSG criteria	Biological treatment: 59.4%; conventional treatment: 40.6%	Illumina MiSeq	V3–V4 regions of the 16S rRNA gene	Greengenes	Shannon
Min 2023	Korea	CC	SpA: 33; HC: 20	42.3 ± 12.4	90.9	2009 ASAS criteria	TNF inhibitor: 51.5%, NSAIDs: 78.8%	Illumina MiSeq	V3–V4 regions of the 16S rRNA gene	SILVA	Observed OTUs, Chao1
Oprea 2022	Romania	CC	AS: 25; HC: 16	49	80.0	Modified New York criteria	NR	Ion Torrent PGM	V2–V9 regions of the 16S rRNA gene	MicroSEQ ID, Greengenes	Chao1, Shannon
Regner 2018	USA	CC	axSpA: 6; HC: 15	34.4 ± 3.3	83.0	2009 ASAS criteria	TNF inhibitor: 83%; prednisone: 17%	Illumina MiSeq	V3–V4 regions of the 16S rRNA gene	SILVA	Shannon, Chao1
Song 2022	China	RC	AS: 62; HC: 62	42.0 ± 13.7	67.7	New York criteria	NSAIDs: 38.7%; steroid hormone: 3.2%; immunosuppressant: 14.5%; biological agents: 16.1%	Illumina MiSeq	V3–V4 regions of the 16S rRNA gene	SILVA	Chao1, ACE
Stoll 2023	UK	CC	AS: 29; HC: 43	45.3 ± 7.1	75.9	Modified New York criteria	NSAIDs: 55.2%; DMARDs: 55.2%; TNF-inhibiting mAb: 37.9%; etanercept: 17.2%; corticosteroids: 10.3%; secukinumab: 3.4%; Methotrexate: 3.4%	Illumina MiSeq	V4 region of the 16S rRNA gene	SILVA	Chao1, Shannon
Sun 2021	China	PC	AS: 9; HC: 9	41.89 ± 7.29	88.9	New York criteria	NR	Illumina platform implied by paired-end sequencing	16S rRNA (specific hypervariable region not stated)	Greengenes	Shannon, Observed OTUs
Tito 2017	Belgium	CC	AS: 27; HC: 15	NR	NR	ASAS criteria	NR	Illumina MiSeq	V4 region of the 16S rRNA gene	Greengenes	Observed OTUs, Shannon
Wang 2025	China	PC	AS: 10; HC: 10	35.20 ± 7.05	70.0	1984 New York criteria	NSAIDs (all patients)	NovaSeq 6000	V3–V4 regions of the 16S rRNA gene	SILVA	ACE, Chao1, Shannon, Simpson, Coverage
Yang 2025	China	CC	AS: 30; HC: 25	18–45	NR	1984 New York criteria	Not specified for other treatments.	NovaSeq PE250 platform	V3–V4 regions of the 16S rRNA gene	NA	Chao1, Observed OTUs
You 2023	China	CC	AS: 40; HC: 40	33.7 ± 10.6	87.5	Modified New York criteria	NR	Illumina MiSeq	V3–V4 regions of the 16S rRNA gene	SILVA	ACE, Chao1, Shannon, Simpson
Yu 2024	China	PC	AS: 30; HC: 30	35.33 ± 8.75	50.0	2010 Guidelines of the Rheumatology Branch of Chinese Medical Association	NR	Illumina MiSeq/HiSeq 2500	16S rDNA (region not specified)	SILVA	Shannon, Simpson, ACE, Chao1
Yuan 2024	China	PC	AS: 13; HC: 13	41.46 ± 12.67	NR	NR	NR	Illumina	V4 region of the 16S rRNA gene	NA	Observed species, ACE, Chao1, Shannon, Simpson
Zhang 2019	China	CC	AS: 103; HC: 104	33.29 ± 11.66	82.5	Modified New York criteria	NSAIDs: 68.9%; biological agents: 44.7%; DMARDs: 34.0%	Illumina MiSeq	V3–V4 regions of the 16S rRNA gene	NA	Chao1, Shannon
Zhang 2020	China	PC	AS: 20; HC: 19	33.18 ± 4.23	100.0	1984 modified New York criteria	anti-TNF-α (adalimumab)	Illumina HiSeq	V3–V4 regions of the 16S rRNA gene	SILVA	Chao1, ACE

NR, not reported; PC, prospective cohort; CC, case–control; RC, retrospective cohort; axSpA, axial spondyloarthritis; AS, ankylosing spondylitis; NSAIDs, nonsteroidal anti-inflammatory drugs; DMARDs, disease-modifying antirheumatic drugs; HC, healthy control.

**Table 2:** Newcastle–Ottawa score for risk-of-bias assessment of the included studies.

Study	Population			Comparability		Outcome		Score		Evaluation
	1	2	3	4		1	2	3		
Berland 2023	1	1	1	1	2	1	1	0	8	Good
Breban 2017	1	0	1	1	2	1	1	0	7	Good
Chen 2019	1	1	1	1	2	1	1	0	8	Good
Chen 2021	1	1	1	1	2	1	1	0	8	Good
Costello 2015	1	0	1	1	2	1	1	0	7	Good
Dai 2022	1	1	1	1	2	1	1	0	8	Good
Gill 2022	1	1	1	1	2	1	1	0	8	Good
Li 2019	1	1	1	1	2	1	1	0	8	Good
Li 2023	1	1	1	0	2	1	1	0	7	Good
Liu 2020	1	0	1	1	2	1	1	0	7	Good
Marquez-Ortiz 2023	1	1	1	1	2	1	1	0	8	Good
Min 2023	1	1	1	0	2	1	1	0	7	Good
Oprea 2022	1	1	1	1	2	1	1	0	8	Good
Regner 2018	1	0	1	1	2	1	1	0	7	Good
Song 2022	1	1	1	1	2	1	1	0	8	Good
Stoll 2023	1	1	1	1	2	1	1	0	8	Good
Sun 2021	1	0	1	1	2	1	1	0	7	Good
Tito 2017	1	1	0	1	2	1	1	0	7	Good
Wang 2025	1	0	1	1	2	1	1	0	7	Good
Yang 2025	1	1	1	1	2	1	1	0	8	Good
You 2023	1	1	1	1	2	1	1	0	8	Good
Yu 2024	1	1	1	1	2	1	1	0	8	Good
Yuan 2024	1	0	1	1	2	1	1	0	7	Good
Zhang 2019	1	1	1	1	2	1	1	0	8	Good
Zhang 2020	1	1	1	1	2	1	1	0	8	Good

### α-Diversity

Observed richness, Chao1, and ACE primarily reflect microbial richness (or richness estimators), whereas Shannon and Simpson reflect diversity incorporating evenness. Because these indices can be reported on different scales across studies, all α-diversity outcomes were synthesized using SMDs.

### Observed richness

**Figure 2:**
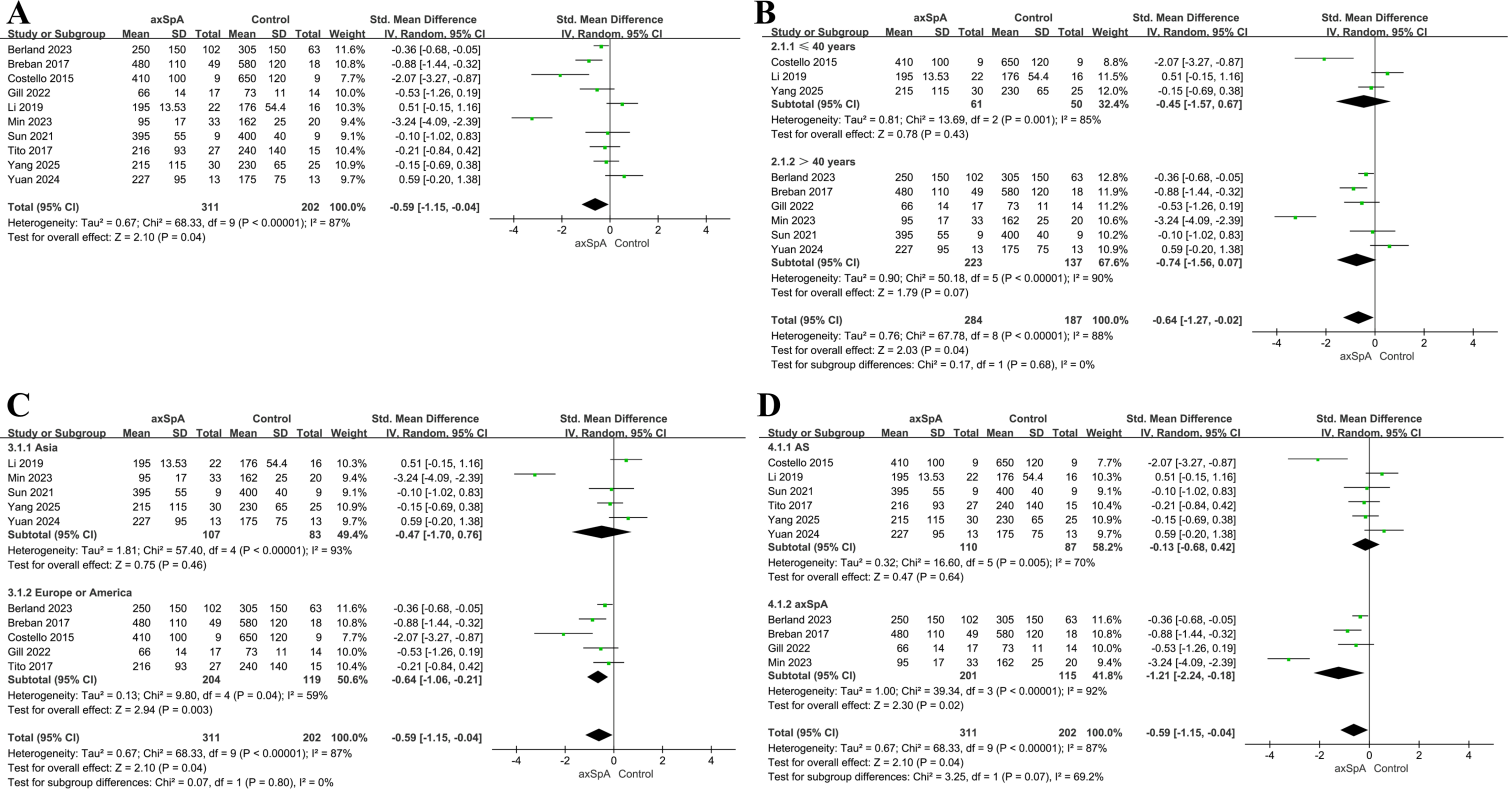
Forest plots of the Observed richness in patients with axSpA versus healthy controls. **(A)** Overall pooled effect. **(B)** Subgroup analysis by age. **(C)** Subgroup analysis by geographic region. **(D)** Subgroup analysis by disease phenotype.

Ten studies including 311 patients and 202 controls reported the Observed richness index (60, 23–31). Across studies, the “Observed” metric was variably reported as observed OTUs, observed species, observed richness, or simply observed; these measures were harmonized as OTU/feature-based observed richness when derived from bacterial feature tables. Overall, microbial richness was significantly lower in patients with axSpA than in controls (SMD = −0.59, 95% CI = −1.15 to −0.04, *P* = 0.04; *I^2^* = 87%; **Figure 2A**). In sensitivity analysis excluding the whole-metagenome shotgun study (Berland 2023) and the study with an unclear definition of “Observed” (Gill 2022), the pooled estimate remained directionally consistent but was no longer statistically significant (SMD = −0.66, 95% CI = −1.45 to 0.13, *P* = 0.10; I² = 90%). In leave-one-out analyses, the pooled effect remained directionally consistent, with SMDs ranging from −0.74 to −0.30 (**Supplementary Figure 1A**), suggesting that the overall association was not dependent on any single study.

In age-stratified analyses, a non-significant trend toward lower richness in patients than in controls was observed among participants aged ≤40 years (three studies, 61 patients, 50 controls; SMD = −0.45, 95% CI = −1.57 to 0.67, *P* = 0.43; *I^2^* = 85%; **Figure 2B**). A similar non-significant trend was observed among participants aged >40 years (six studies, 223 patients, 137 controls; SMD = −0.74, 95% CI = −1.56 to 0.07, *P* = 0.07; *I^2^* = 90%). No significant interaction between age groups was observed (*P* = 0.68). One study that did not report age was excluded from the age subgroup analysis.

In the subgroup analysis by geographic region, studies conducted in Asia (five studies, 107 patients, 83 controls) revealed no significant difference in richness between patients and controls (SMD = −0.47, 95% CI = −1.70 to 0.76, *P* = 0.46; *I^2^* = 93%; **Figure 2C**), whereas studies from Europe and North America (five studies, 204 patients, 119 controls) identified significantly lower richness in patients (SMD = −0.64, 95% CI = −1.06 to −0.21 *I^2^* = 59%; *P* = 0.003). The test for subgroup differences between regions was not statistically significant (*P* = 0.80).

When stratified by phenotype, AS-only studies (six studies, 110 patients, 87 controls) identified no significant reduction in richness among patients (SMD = −0.13, 95% CI = −0.68 to 0.42, *P* = 0.64; *I^2^* = 70%; **Figure 2D**), whereas studies including broader axSpA populations (four studies, 201 patients, 115 controls) revealed significantly lower richness among patients (SMD = −1.21, 95% CI = −2.24 to −0.18, *P* = 0.02; *I^2^* = 92%). The test for subgroup differences between phenotypes was not statistically significant (*P* = 0.07).

### Shannon index

**Figure 3:**
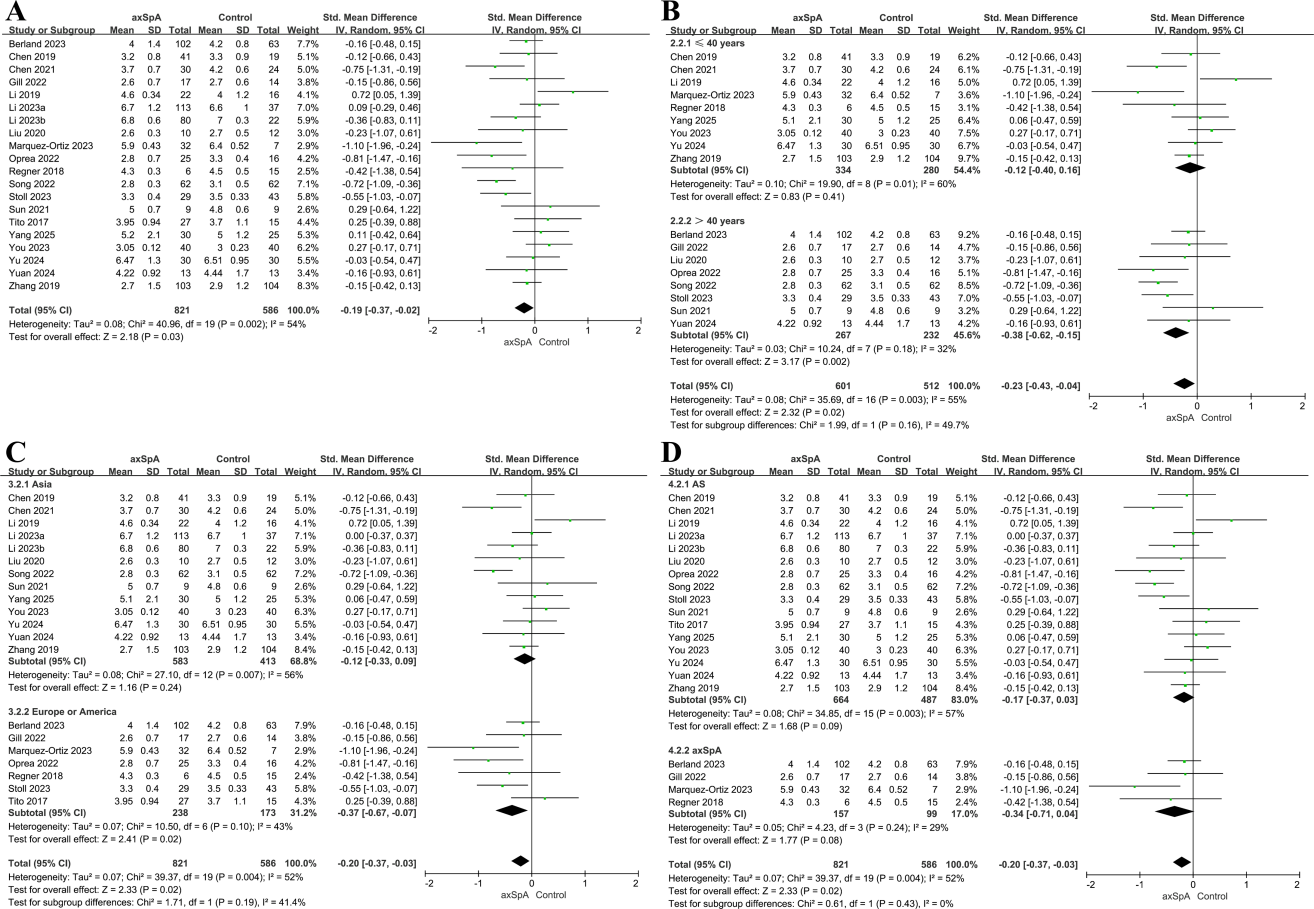
Forest plots of the Shannon index in patients with axSpA versus healthy controls. **(A)** Overall pooled effect. **(B)** Subgroup analysis by age. **(C)** Subgroup analysis by geographic region. **(D)** Subgroup analysis by disease phenotype.

Twenty studies involving 821 patients and 586 controls reported Shannon index (60, 25, 26, 28, 32–40). Overall, microbial diversity was lower in patients with axSpA (SMD = −0.19, 95% CI = −0.37 to −0.02, *P* = 0.03; *I^2^* = 54%; **Figure 3A**). In sensitivity analysis excluding the two whole-metagenome shotgun studies (Berland 2023 and Li 2023), the pooled estimate remained directionally consistent but was no longer statistically significant (SMD = −0.21, 95% CI = −0.42 to 0.01, *P* = 0.06; I² = 58%). In leave-one-out analyses, the pooled effect remained directionally consistent (SMD range, −0.24 to −0.16, **Supplementary Figure 1B**), indicating that no single study materially drove the overall association.

In age-stratified analyses, no difference in Shannon index was noted between patients with axSpA and controls among participants aged ≤40 years (nine studies, 334 patients, 280 controls; SMD = −0.12, 95% CI = −0.40 to 0.16, *P* = 0.41; *I^2^* = 60%; **Figure 3B**), whereas a significant reduction among patients was noted among participants aged >40 years (eight studies, 267 patients, 232 controls; SMD = −0.38, 95% CI = −0.62 to −0.15, *P* = 0.002; *I^2^* = 32%). The difference between age subgroups was not significant (*P* = 0.16). Three studies that did not report age were excluded from this analysis.

Regional analysis identified no significant difference in Shannon index between patients and controls in Asian studies (13 studies, 583 patients, 413 controls; SMD = −0.12, 95% CI = −0.33 to 0.09, *P* = 0.24; *I^2^* = 56%; **Figure 3C**), whereas European/North American studies (seven studies, 238 patients, 173 controls) reported significantly lower diversity in patients (SMD = −0.37, 95% CI = −0.67 to −0.07, *P* = 0.02; *I^2^* = 43%). The regional difference was not statistically significant (*P* = 0.19).

Phenotype-stratified analysis showed no significant reduction in Shannon index in either patients with AS (16 studies, 664 patients, 487 controls; SMD = −0.17, 95% CI = −0.37 to 0.03, *P* = 0.09; *I^2^* = 57%; **Figure 3D**) or those with axSpA (four studies, 157 patients, 99 controls; SMD = −0.34, 95% CI = −0.71 to 0.04, *P* = 0.08; *I^2^* = 29%), with no significant difference between subgroups (*P* = 0.43).

### Simpson index

**Figure 4:**
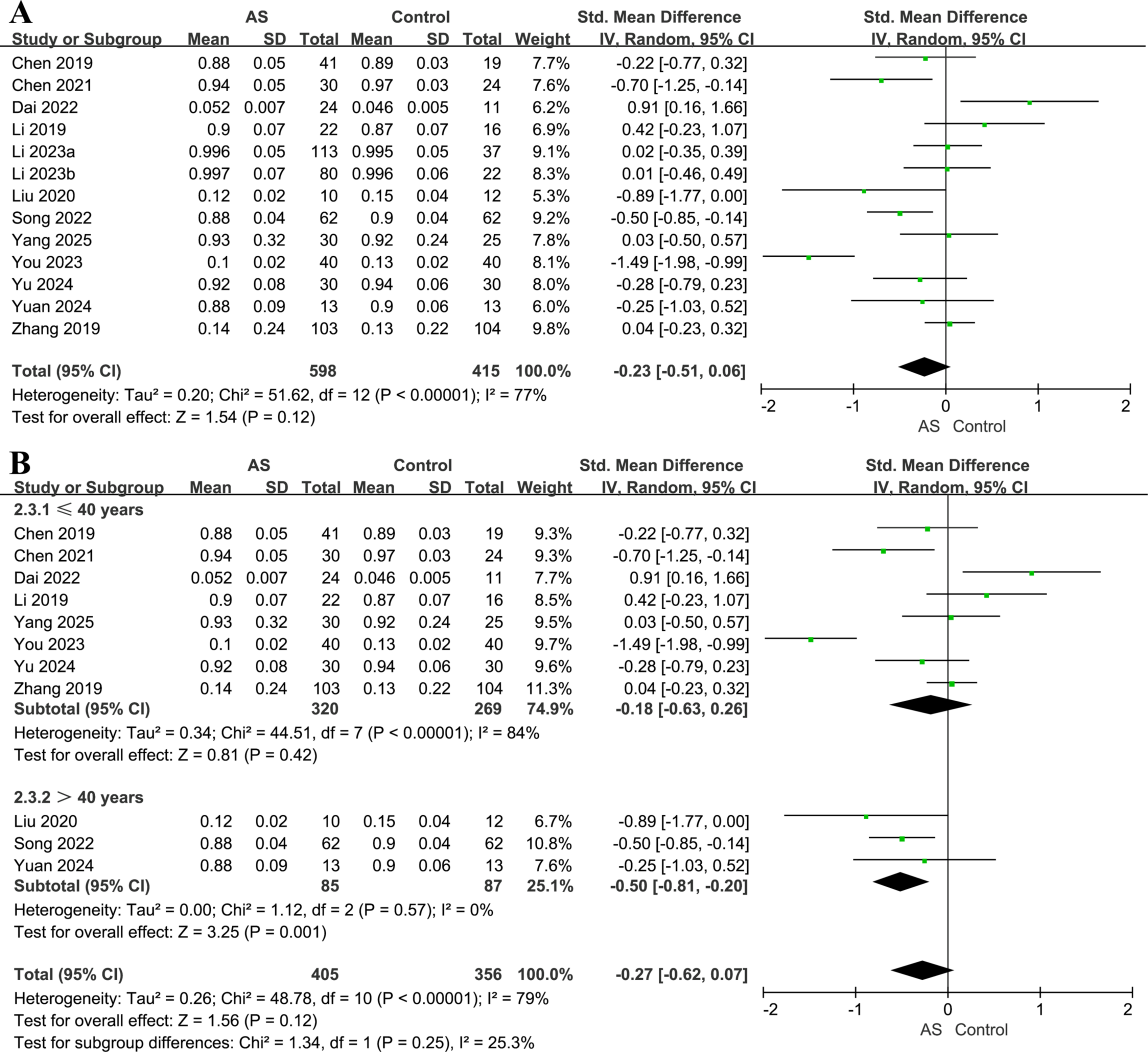
Forest plots of the Simpson index in patients with AS versus healthy controls. **(A)** Overall pooled effect. **(B)** Subgroup analysis by age.

Thirteen studies including 598 patients and 415 controls reported the Simpson index (22, 26, 30–35, 39, 41–43). Overall, no significant difference in this index was observed between patients and controls (SMD = −0.23, 95% CI = −0.51 to 0.06, *P* = 0.12; *I^2^* = 77%; **Figure 4A**). In sensitivity analysis excluding the whole-metagenome shotgun study (Li 2023), the pooled estimate remained unchanged (SMD = −0.27, 95% CI = −0.62 to 0.07, *P* = 0.12; I² = 79%). Leave-one-out analyses showed consistent directionality, with pooled SMDs ranging from −0.30 to −0.12 (**Supplementary Figure 1C**), supporting the robustness of the overall estimate.

Age-stratified analyses identified no significant difference in the Simpson index between patients and controls among participants aged ≤40 years (eight studies, 320 patients, 269 controls; SMD = −0.18, 95% CI −0.63 to 0.26, *P* = 0.42; *I^2^* = 84%; **Figure 4B**). By contrast, among participants aged >40 years (three studies, 85 patients, 87 controls), a statistically significant reduction in microbial evenness was noted among patients (SMD = −0.50, 95% CI = −0.81 to −0.20, *P* = 0.001; I² = 0%), indicating that reductions in microbial evenness in AS are largely confined to older adults. All included studies were conducted in China, and only patients with AS were enrolled, preventing further subgroup analyses.

### Chao1 index

**Figure 5:**
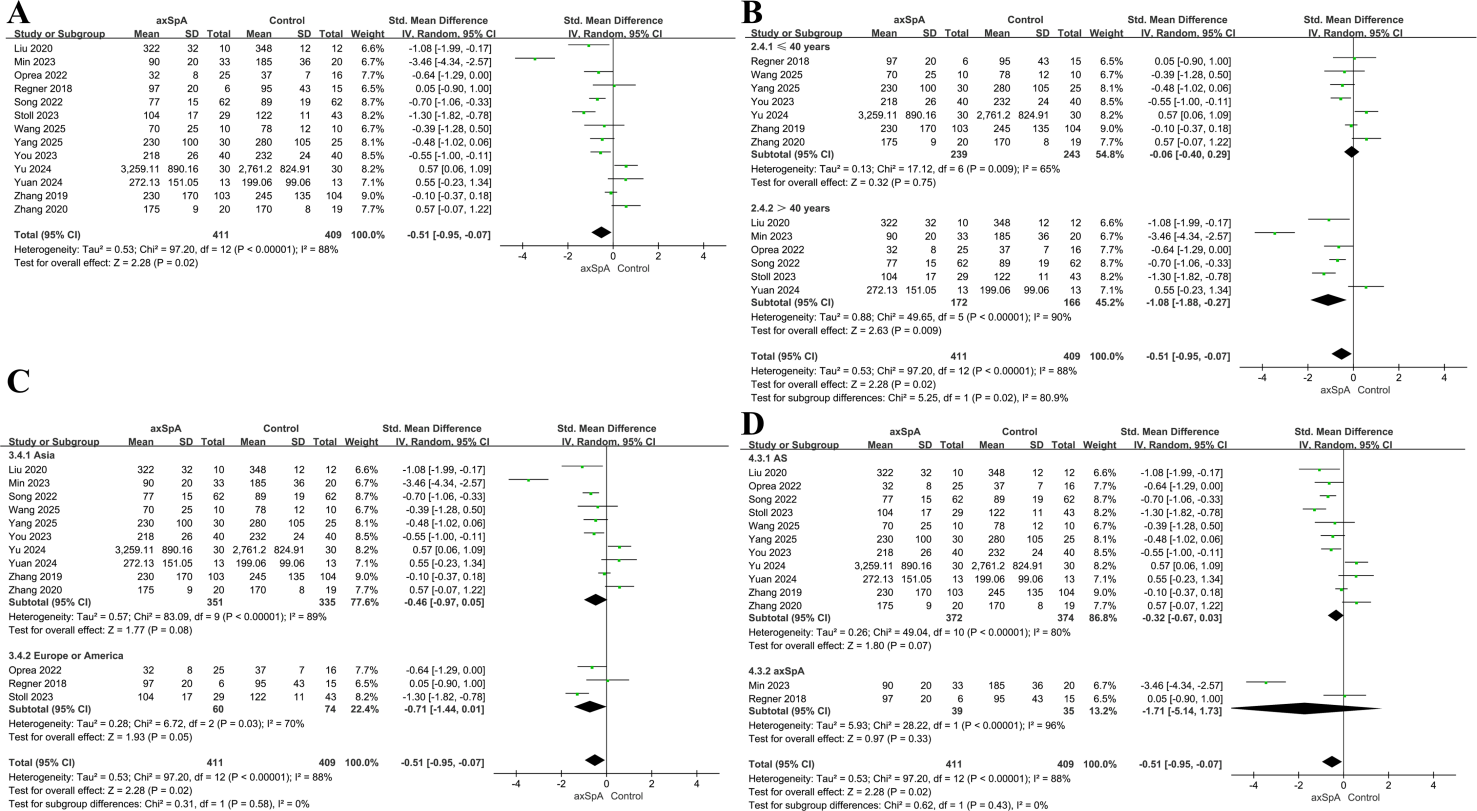
Forest plots of the Chao1 index in patients with axSpA versus healthy controls. **(A)** Overall pooled effect. **(B)** Subgroup analysis by age. **(C)** Subgroup analysis by geographic region. **(D)** Subgroup analysis by disease phenotype.

Thirteen studies including 411 patients and 409 controls reported the Chao1 index (27, 30, 31, 35, 37–40, 42–45). Overall, microbial richness was significantly lower in patients with axSpA (SMD = −0.51, 95% CI = −0.95 to −0.07, *P* = 0.02; *I^2^* = 88%; **Figure 5A**). In leave-one-out analyses, the pooled SMD remained directionally consistent (range, −0.61 to −0.30, **Supplementary Figure 1D**), suggesting that the overall association was not driven by a single study.

Among participants aged >40 years (six studies, 172 patients, 166 controls), richness was significantly lower among patients (SMD = −1.08, 95% CI = −1.88 to −0.27, *P* = 0.009; *I^2^* = 90%; **Figure 5B**), whereas no difference was observed among participants aged ≤40 years (seven studies, 239 patients, 243 controls; SMD = −0.06, 95% CI = −0.40 to 0.29, *P* = 0.75; *I^2^* = 65%). The subgroup difference was statistically significant (*P* = 0.02).

Regionally, Asian studies (10 studies, 351 patients, 335 controls) recorded a non-significant trend toward reduced Chao1 values in patients (SMD = −0.46, 95% CI = −0.97 to 0.05, *P* = 0.08; *I^2^* = 89%; **Figure 5C**), whereas a non-significant trend toward lower Chao1 was observed in European/North American studies (three studies, 60 patients, 74 controls; SMD = −0.71, 95% CI = −1.44 to 0.01, *P* = 0.05; *I^2^* = 70%). No significant difference between subgroups was found (*P* = 0.58).

Phenotype-stratified analysis showed a non-significant trend toward lower richness in AS patients (eleven studies, 372 patients, 374 controls; SMD = −0.32, 95% CI −0.67 to 0.03, *P* = 0.07; *I^2^* = 80%; **Figure 5D**) and no significant difference in patients with axSpA (two studies, 39 patients, 35 controls; SMD = −1.71, 95% CI = −5.14 to 1.73, *P* = 0.33; *I^2^* = 96%). No significant difference between subgroups was found (*P* = 0.43).

### ACE index

**Figure 6:**
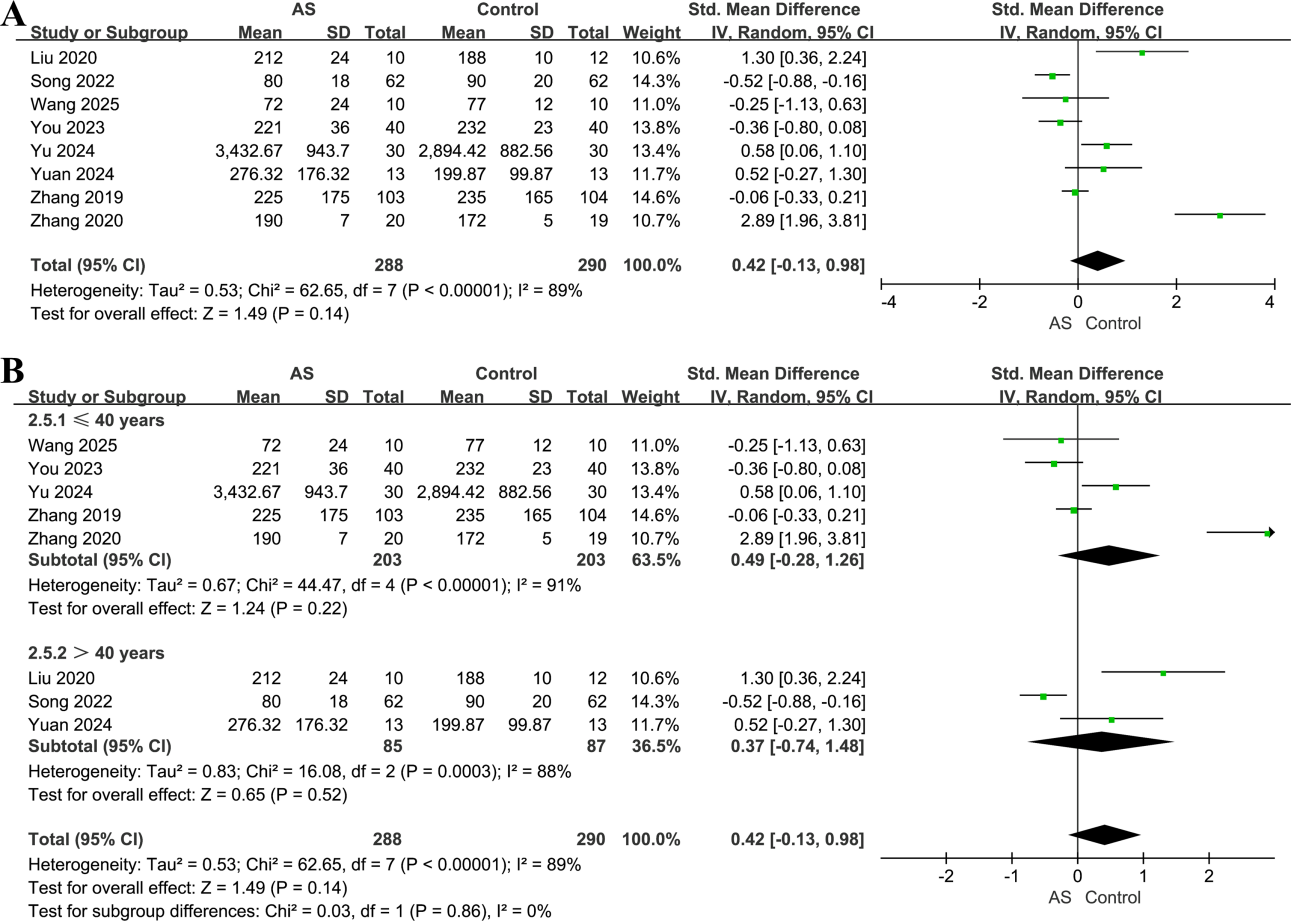
Forest plots of the ACE index in patients with AS versus healthy controls. **(A)** Overall pooled effect. **(B)** Subgroup analysis by age.

Eight studies including 288 patients and 290 controls reported the ACE index (22, 31, 35, 39, 42–45). Overall, no significant difference in this index was found between patients and controls (SMD = 0.42, 95% CI = −0.13 to 0.98, *P* = 0.14; *I^2^* = 89%; **Figure 6A**). Leave-one-out analyses yielded pooled SMDs ranging from 0.10 to 0.62 (**Supplementary Figure 1E**), with wide confidence intervals across iterations, consistent with the overall non-significant result.

No differences between patients and controls were observed among participants aged ≤40 years (five studies, 203 patients, 203 controls; SMD = 0.49, 95% CI = −0.28 to 1.26, *P* = 0.22; *I^2^* = 91%; **Figure 6B**) or those aged >40 years (three studies, 85 patients, 87 controls; SMD = 0.37, 95% CI = −0.74 to 1.48, *P* = 0.52; *I^2^* = 88%). The difference between age subgroups was not statistically significant (*P* = 0.86). All studies were conducted in China, and only patients with AS were included, preventing further subgroup analyses.

### Publication bias

Funnel plots were constructed for α-diversity outcomes with ≥10 studies. The plots for Observed richness, Shannon, and Simpson were broadly symmetric (**Supplementary Figures 2A–C**). Mild asymmetry was observed for Chao1 (**Supplementary Figure 2D**). Overall, publication bias was considered low to moderate.

### Taxonomic composition (phylum level)

Phylum-level relative abundance data were synthesized descriptively because many studies did not report measures of dispersion required for meta-analysis. Specifically, most studies provided only point estimates of relative abundance (%) without SD/SE/IQR (and related information needed to derive study-level variances), precluding a valid quantitative meta-analysis of proportions. At the phylum level, data from 14 studies (60, 23, 26–28, 30–32, 33, 35, 42–45) revealed broadly similar proportions of Firmicutes between patients with axSpA and controls (51.35% vs. 51.22%; **Table 3**). However, patients exhibited a reduction in Bacteroidota (33.00% vs. 36.23%) and an increase in Proteobacteria in patients with axSpA (8.43% vs. 5.96%), often associated with inflammation. Eleven studies (23, 26, 27, 30–33, 35, 42–44) also reported a slightly reduced abundance of Actinobacteriota in patients (3.82% vs. 4.86%). To help visualize the direction and heterogeneity of study-level differences, we additionally present study-level paired comparisons (HC vs. axSpA) for major phyla in **Supplementary Figure 3**.

**Table 3:** Relative abundance of major gut microbial phyla in patients with axSpA and healthy controls.

Phylum	axSpA [%, mean (range)]	Healthy control [%, mean (range)]	Result
Firmicutes	51.35 (29–75)	51.22 (29–77)	No change
Bacteroidota	33.00 (10–55)	36.23 (9–69)	Decline
Proteobacteria	8.43 (4.1–20)	5.96 (0.5–18)	Increase
Actinobacteriota	3.82 (0.1–13)	4.86 (0.2–18)	Slightly decline

## Discussion

This meta-analysis illustrated that patients with axSpA exhibit overall gut microbiota alterations compared with healthy controls. Across 25 studies involving 1639 participants, pooled analyses suggested that α-diversity tended to be lower in axSpA, with the most consistent signals observed for richness/diversity metrics (Observed richness, Shannon, and Chao1), whereas Simpson and ACE showed no clear overall differences. Across prespecified sensitivity and leave-one-out analyses, effect directions were generally consistent, although the overall differences for Observed richness and Shannon became non-significant after excluding non-comparable datasets. The more pronounced dysbiosis in older patients might be explained by several factors, including a longer disease duration, with advanced axSpA and more severe inflammation perpetuating microbial alterations; greater cumulative exposure to medications such as NSAIDs and biologics, both of which have been found to reduce microbial diversity and alter taxonomic composition; and age-related gut physiological changes, including reduced motility, impaired immune surveillance, and altered bile acid metabolism (46, 47).

Geographic variation was also suggested Subgroup analyses indicated that European and North American cohorts exhibited significantly lower α-diversity, whereas studies from Asia generally did not report significant differences. These discrepancies likely reflect environmental and dietary factors. Western diets, typically high in fat and protein but low in fiber, promote the expansion of pro-inflammatory taxa such as Proteobacteria. By contrast, Asian diets often contain more plant-based fiber and fermented foods, which might foster beneficial taxa such as Bifidobacterium (48, 49). At the phylum level, patients with axSpA exhibited decreases in the abundance of Bacteroidota and Actinobacteriota alongside an increase in that of Proteobacteria, suggesting a pro-inflammatory microbial environment. Collectively, these findings suggest that gut microbial dysbiosis may be associated with axSpA and could contribute to disease pathophysiology, although heterogeneity and methodological variability warrant cautious interpretation.

The observed reduction in microbial diversity implies a less stable and functionally restricted gut ecosystem. The loss of microbial richness might weaken colonization resistance and facilitate the expansion of pathobionts, particularly Proteobacteria, which are closely linked to inflammation (50). In particular, the reduced abundance of *F. prausnitzii*, a key butyrate producer, might decrease intestinal butyrate levels. Butyrate plays a central role in maintaining epithelial barrier integrity by upregulating tight junction proteins (*e.g.*, occludin) and regulating T cell differentiation by promoting regulatory T cells while inhibiting Th17 cells (34). SCFAs, including acetate, propionate and butyrate, may further modulate mucosal immunity through GPCR signaling and histone deacetylase inhibition, thereby supporting Treg differentiation and restraining Th17-skewed responses (51, 52). Beyond inflammation, gut dysbiosis may also be indirectly related to the “bone paradox” of axSpA, in which bone loss/osteoporosis coexists with pathological new bone formation such as syndesmophytes. This association may reflect microbiota-related immune and metabolic changes that could influence osteoclast–osteoblast coupling; however, direct evidence linking these pathways to structural outcomes in axSpA remains limited (53).

Conversely, expansion of *Escherichia* and *Shigella*, two genera within Proteobacteria, might enhance lipopolysaccharide (LPS) production, activate the TLR4–NF-κB pathway, and drive the release of pro-inflammatory cytokines such as IL-17 and TNF-α (54). These changes collectively compromise intestinal barrier function, facilitate microbial translocation, and sustain systemic inflammation at entheseal and articular sites, thereby strengthening the biological basis of the gut–joint axis (55). Across phenotype-stratified analyses, the direction of effects was broadly similar, but several comparisons did not reach statistical significance, likely reflecting limited study numbers and heterogeneity. This finding aligns with prior reports of reduced SCFA-producing taxa such as *F. prausnitzii* in axSpA and experimental studies indicating that supplementation with butyrate or *F. prausnitzii* can downregulate IL-17A and upregulate IL-10 (27).

Additional pathways may link dysbiosis to axSpA. Molecular mimicry between microbial and host antigens could promote autoreactive T cell responses at entheseal sites (56). Therapeutic interventions appear to modulate the microbiome; for instance, TNF inhibitors have been demonstrated to partially restore the microbial composition and metabolite profiles, with a prospective study in AS demonstrating normalization of taxa such as *Ruminococcus gnavus* and *Bacteroides uniformis* alongside metabolomic changes (57).

Lifestyle and clinical factors are also likely to influence the microbiome in axSpA. Reduced physical activity, common among patients with chronic pain and stiffness, can further exacerbate dysbiosis. Medications also play a role; specifically, long-term NSAID or antibiotic use can reduce microbial diversity, whereas biologics such as TNF inhibitors can exert an indirect restorative effect through immune modulation. Recent reviews also suggested that ultra-processed foods decrease microbial diversity, reduce beneficial taxa, and impair gut barrier function, which could be relevant to axSpA (58).

Our findings carry potential clinical implications for microbiota-targeted therapies; however, these suggestions are hypothesis-generating given the observational nature of the evidence and residual heterogeneity. First, older patients (>40 years) might benefit from dietary and microbiota-supportive strategies to restore microbial diversity and butyrate production. Second, patients in Western countries might benefit from dietary modification strategies, such as increasing fiber intake and reducing processed foods, to counteract Proteobacteria expansion. Asian patients could require population-specific probiotics to enhance microbial stability. Third, probiotics and prebiotics might help restore microbial balance and dampen inflammation. Although evidence remains limited, some small human studies reported improvements in CRP levels and quality of life in patients with axSpA receiving probiotics (59). Fecal microbiota transplantation is also under investigation, with one randomized controlled trial registered in axSpA. Moreover, metabolomic data suggest that modifying microbial metabolites—whether through diet, microbiota-based therapies, or biologics—might influence pathways involved in cartilage and bone destruction, such as purine metabolism and linoleic acid derivatives. Together, these results provide a rationale for integrating microbiome profiling into axSpA management. Such strategies could enable early risk stratification, support precision nutrition and microbiota-based interventions, ultimately contributing to long-term disease control.

This study had several limitations. First, most of the included studies were conducted in China (n = 15), which might limit the generalizability of our findings to other populations, such as those in Africa and South America. Second, substantial heterogeneity was observed across studies, which could be partly explained by methodological differences. For instance, variations in 16S rRNA sequencing regions (*e.g.*, V3–V4 vs. V4) and taxonomic reference databases (*e.g.*, SILVA vs. Greengenes) might have influenced the estimation of microbial diversity. Moreover, rarefaction, sequencing-depth thresholds, and the 16S region were inconsistently reported, precluding reliable stratified sensitivity analyses. Third, the majority of included studies adopted a cross-sectional design, precluding causal inference between gut microbiota alterations and axSpA onset or progression. Finally, only a few studies reported comprehensive metadata, such as dietary patterns, physical activity, medication history, or gut barrier function markers, which restricted our ability to conduct more refined subgroup or adjusted analyses. Future studies should address these limitations by enrolling more diverse populations, applying standardized sequencing protocols, and collecting longitudinal, metadata-rich datasets to better clarify the causal pathways linking microbiota to axSpA.

## Conclusion

This meta-analysis suggests an association between axSpA and gut microbiota dysbiosis, characterized by lower α-diversity and compositional shifts at the phylum level. However, substantial heterogeneity and methodological variability across studies, together with predominantly cross-sectional designs and limited functional readouts, warrant cautious interpretation. Future longitudinal, multi-omics, and interventional studies are needed to clarify causality and evaluate microbiota-targeted strategies in axSpA.

## Data Availability

The original contributions presented in the study are included in the article/**Supplementary Material**. Further inquiries can be directed to the corresponding author/s.
