# Supplementary material for: Age- and region-specific gut microbiota dysbiosis in axial spondyloarthritis: a systematic review and meta-analysis
Source: Front Immunol. 2026 Mar 16;17:1736358. doi: 10.3389/fimmu.2026.1736358 (PMC13033507; doi:10.3389/fimmu.2026.1736358)
Supplement: Supplementary file 1 [file DataSheet1.pdf]

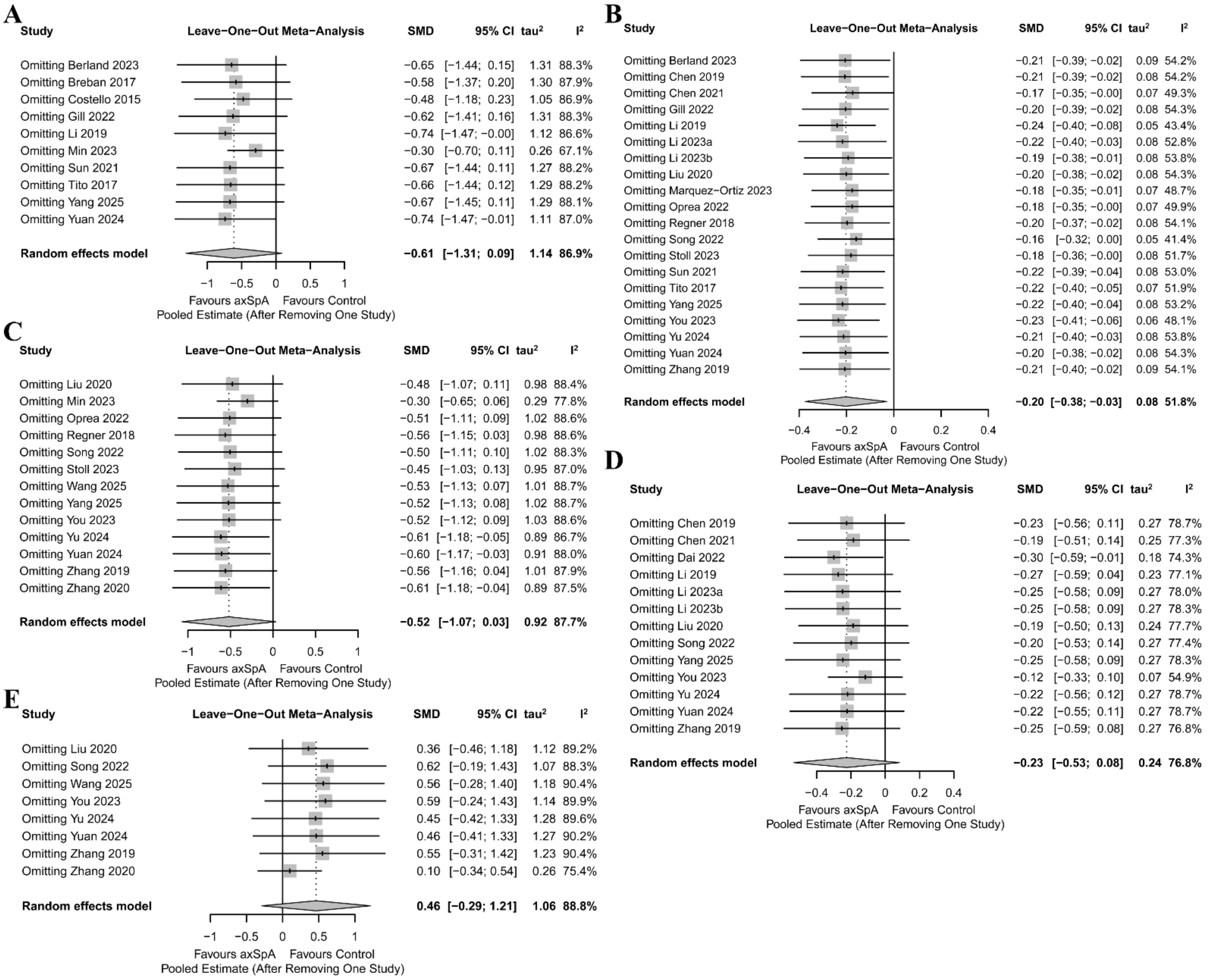

Supplementary Figure 1 Leave-one-out sensitivity analyses for α-diversity indices.(A) Observed richness index; (B) Shannon index; (C) Simpson index; (D) Chao1 index; and (E) ACE index.

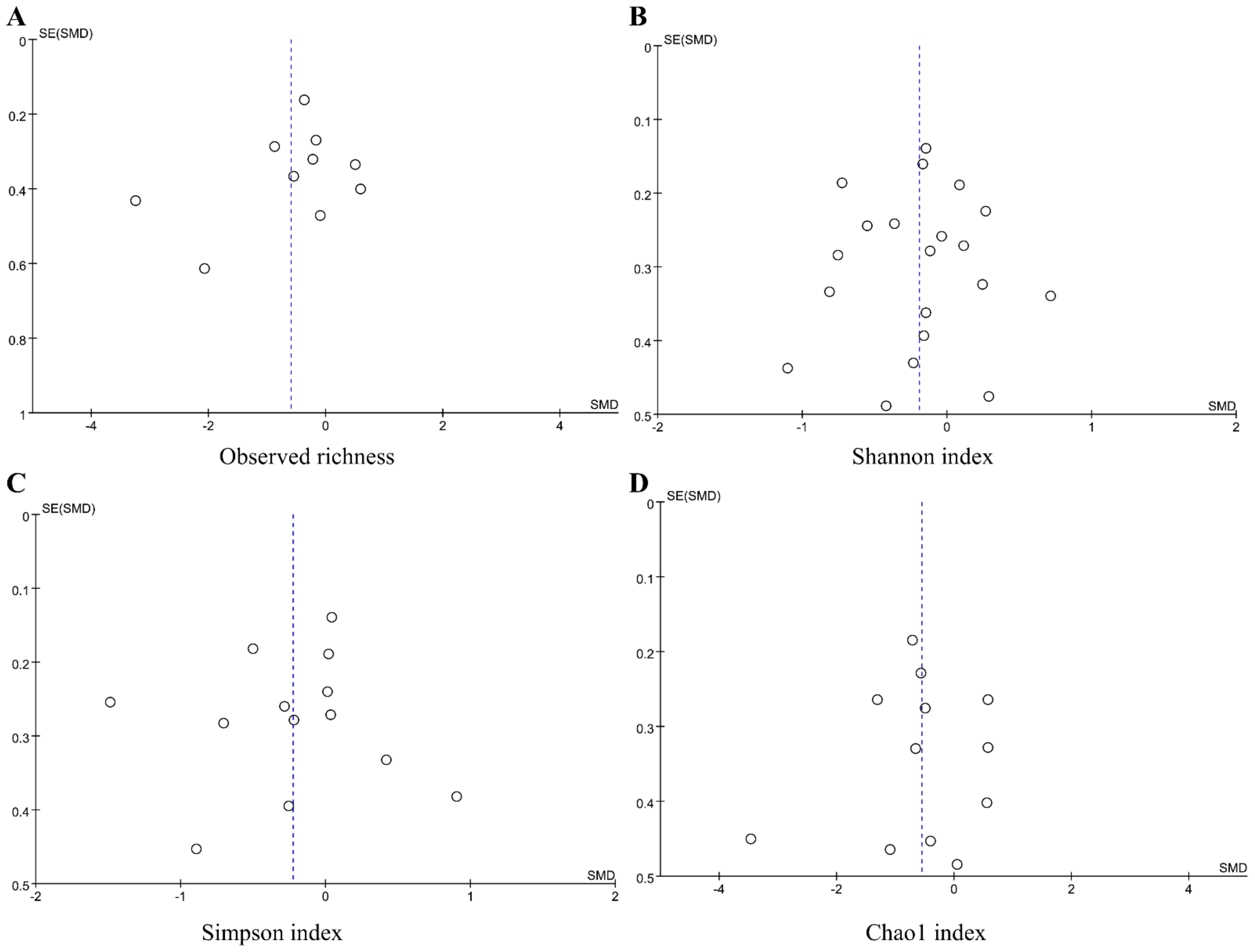

Supplementary Figure 2 Funnel plot for estimating the potential publication biases underlying the meta-analyses of the association between patients with axSpA/AS versus controls. (A) Observed richness index. (B) Shannon index. (C) Simpson index. (D) Chao1 index.

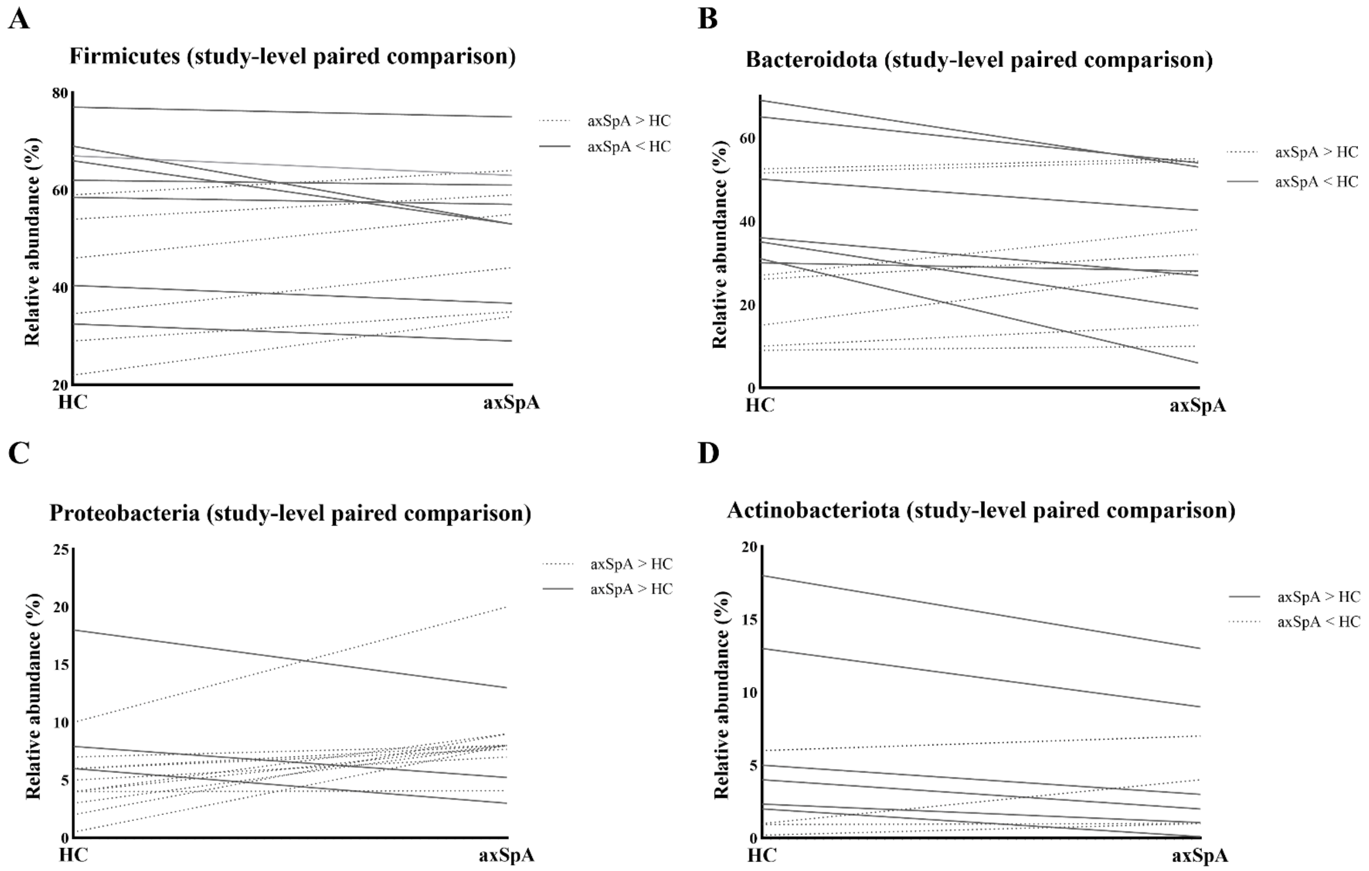

Supplementary Figure 3 Study-level paired comparisons of major gut microbial phyla between axSpA and healthy controls. (A) Firmicutes, (B) Bacteroidota, (C) Proteobacteria, and (D) Actinobacteriota.
