# Supplementary material for: Age- and region-specific gut microbiota dysbiosis in axial spondyloarthritis: a systematic review and meta-analysis
Source: Front Immunol. 2026 Mar 16;17:1736358. doi: 10.3389/fimmu.2026.1736358 (PMC13033507; doi:10.3389/fimmu.2026.1736358)
Supplement: Supplementary file 2 [file Table1.docx]

**Supplementary Table 1 The search strategy of each database**

| **Database** | **Search strategy** | **Results** |
| --- | --- | --- |
| **PubMed** | #1 (("Axial Spondyloarthritis"[MeSH Terms]) OR ("Spondylitis, Ankylosing"[MeSH Terms])) OR (("Spondyloarthriti*, Axial"[Title/Abstract] OR "Ankylosing Spondylitis"[Title/Abstract] OR AxSpA[Title/Abstract] OR "Ankylosing Spondylarthriti*"[Title/Abstract] OR "Spondylarthriti*, Ankylosing"[Title/Abstract] OR "Ankylosing Spondyloarthriti*"[Title/Abstract] OR "Spondyloarthriti*, Ankylosing"[Title/Abstract] OR "Spondylitis Ankylopoietica"[Title/Abstract] OR "Bechterew Disease"[Title/Abstract] OR "Spondyloarthritis Ankylopoietica"[Title/Abstract] OR "non-radiographic axial spondyloarthritis"[Title/Abstract] OR nr-axSpA[Title/Abstract] OR "Undifferentiated Spondylarthropathy"[Title/Abstract])) | 24828 |
|  | #2 ("Gastrointestinal Microbiome"[MeSH Terms]) OR (("Gastrointestinal Microbiomes"[Title/Abstract] OR "Microbiome, Gastrointestinal"[Title/Abstract] OR "GI Microflora*"[Title/Abstract] OR "Microbiome*, GI"[Title/Abstract] OR "GI Microbiome*"[Title/Abstract] OR "Enteric Microbiota*"[Title/Abstract] OR "Microbiota*, Enteric"[Title/Abstract] OR "Gut Microflora"[Title/Abstract] OR "Microflora, Gut"[Title/Abstract] OR "Gastrointestinal Microflora"[Title/Abstract] OR "Microflora, Gastrointestinal"[Title/Abstract] OR "Gastrointestinal Flora"[Title/Abstract] OR "Flora, Gastrointestinal"[Title/Abstract] OR "Gut Flora"[Title/Abstract] OR "Flora, Gut"[Title/Abstract] OR "Gastrointestinal Microbial Communit*"[Title/Abstract] OR "Gut Microbiome*"[Title/Abstract] OR "Microbiome, Gut"[Title/Abstract] OR "Gastrointestinal Microbiota*"[Title/Abstract] OR "Microbiota, Gastrointestinal"[Title/Abstract] OR Microflora*[Title/Abstract] OR "Gut Microbiota*"[Title/Abstract] OR "Microbiota, Gut"[Title/Abstract] OR "Intestinal Microbiome*"[Title/Abstract] OR "Microbiome, Intestinal"[Title/Abstract] OR "Intestinal Microflora"[Title/Abstract] OR "Microflora, Intestinal"[Title/Abstract] OR "Intestinal Flora"[Title/Abstract] OR "Flora, Intestinal"[Title/Abstract] OR "Intestinal Microbiota*"[Title/Abstract] OR "Microbiota, Intestinal"[Title/Abstract] OR "Enteric Bacteria"[Title/Abstract] OR "Bacteria, Enteric"[Title/Abstract] OR "Gastric Microbiome*"[Title/Abstract] OR "Microbiome, Gastric"[Title/Abstract])) | 131341 |
|  | #3 #1 and #2 | 222 |
|  | #4 Animals[Mesh] NOT Humans[Mesh] | 5364162 |
|  | #5 #3 NOT #4 | **208** |
| **Embase** | #1 'axial spondyloarthritis'/exp | 44246 |
|  | #2 'spondylitis, ankylosing'/exp | 37926 |
|  | #3 'spondyloarthriti*, axial':ab,ti OR 'ankylosing spondylitis':ab,ti OR axspa:ab,ti OR 'ankylosing spondylarthriti*':ab,ti OR 'spondylarthriti*, ankylosing':ab,ti OR 'ankylosing spondyloarthriti*':ab,ti OR 'spondyloarthriti*, ankylosing':ab,ti OR 'spondylitis ankylopoietica':ab,ti OR 'bechterew disease':ab,ti OR 'spondyloarthritis ankylopoietica':ab,ti OR 'non-radiographic axial spondyloarthritis':ab,ti OR 'nr axspa':ab,ti OR 'undifferentiated spondylarthropathy':ab,ti | 35041 |
|  | #4 #1 OR #2 OR #3 | 48475 |
|  | #5 'gastrointestinal microbiome'/exp | 138543 |
|  | #6 'gastrointestinal microbiomes':ab,ti OR 'microbiome, gastrointestinal':ab,ti OR 'gi microflora*':ab,ti OR 'microbiome*, gi':ab,ti OR 'gi microbiome*':ab,ti OR 'enteric microbiota*':ab,ti OR 'microbiota*, enteric':ab,ti OR 'gut microflora':ab,ti OR 'microflora, gut':ab,ti OR 'gastrointestinal microflora':ab,ti OR 'microflora, gastrointestinal':ab,ti OR 'gastrointestinal flora':ab,ti OR 'flora, gastrointestinal':ab,ti OR 'gut flora':ab,ti OR 'flora, gut':ab,ti | 7675 |
|  | #7 'gastrointestinal microbial communit*':ab,ti OR 'gut microbiome*':ab,ti OR 'microbiome, gut':ab,ti OR 'gastrointestinal microbiota*':ab,ti OR 'microbiota, gastrointestinal':ab,ti OR microflora*:ab,ti OR 'gut microbiota*':ab,ti OR 'microbiota, gut':ab,ti OR 'intestinal microbiome*':ab,ti OR 'microbiome, intestinal':ab,ti OR 'intestinal microflora':ab,ti OR 'microflora, intestinal':ab,ti OR 'intestinal flora':ab,ti OR 'flora, intestinal':ab,ti OR 'intestinal microbiota*':ab,ti OR 'microbiota, intestinal':ab,ti OR 'enteric bacteria':ab,ti OR 'bacteria, enteric':ab,ti OR 'gastric microbiome*':ab,ti OR 'microbiome, gastric':ab,ti | 142040 |
|  | #8 #5 OR #6 OR #7 | 186552 |
|  | #9 #4 AND #8 | 452 |
|  | #10 #4 AND #8 AND ('article'/it OR 'article in press'/it OR 'editorial'/it OR 'letter'/it OR 'clinical trial'/it) AND [humans]/lim | **177** |
| **Web of Science** | TS=(“Axial Spondyloarthritis” OR “Spondylitis, Ankylosing” OR “Spondyloarthriti*, Axial” OR “Ankylosing Spondylitis” OR AxSpA OR “Ankylosing Spondylarthriti*” OR “Spondylarthriti*, Ankylosing” OR “Ankylosing Spondyloarthriti*” OR “Spondyloarthriti*, Ankylosing” OR “Spondylitis Ankylopoietica” OR “Bechterew Disease” OR “Spondyloarthritis Ankylopoietica” OR "non-radiographic axial spondyloarthritis" OR nr-axSpA OR "Undifferentiated Spondylarthropathy") AND (“Gastrointestinal Microbiome” OR “Gastrointestinal Microbiomes” OR “Microbiome, Gastrointestinal” OR “GI Microflora*” OR “Microbiome*, GI” OR “GI Microbiome*” OR “Enteric Microbiota*” OR “Microbiota*, Enteric” OR “Gut Microflora” OR “Microflora, Gut” OR “Gastrointestinal Microflora” OR “Microflora, Gastrointestinal” OR “Gastrointestinal Flora” OR “Flora, Gastrointestinal” OR “Gut Flora” OR “Flora, Gut” OR “Gastrointestinal Microbial Communit*” OR “Gut Microbiome*” OR “Microbiome, Gut” OR “Gastrointestinal Microbiota*” OR “Microbiota, Gastrointestinal” OR Microflora* OR “Gut Microbiota*” OR “Microbiota, Gut” OR “Intestinal Microbiome*” OR “Microbiome, Intestinal” OR “Intestinal Microflora” OR “Microflora, Intestinal” OR “Intestinal Flora” OR “Flora, Intestinal” OR “Intestinal Microbiota*” OR “Microbiota, Intestinal” OR “Enteric Bacteria” OR “Bacteria, Enteric” OR “Gastric Microbiome*” OR “Microbiome, Gastric”) | 407 |
|  | Quick Filters: Review Article Exclude  Document Types: Article/Clinical Trial Refine | **181** |
| **Cochrane Library** | #1 Title Abstract Keyword=(“Axial Spondyloarthritis” OR “Ankylosing Spondylitis” OR AxSpA OR nr-axSpA OR "non-radiographic axial spondyloarthritis" OR “Bechterew Disease” OR “Undifferentiated Spondylarthropathy” OR "Spondylitis Ankylopoietica" OR "Spondyloarthritis Ankylopoietica") | 3225 |
|  | #2 Title Abstract Keyword=("Gastrointestinal Microbiome" OR "Gut Microbiome" OR "Intestinal Microbiota" OR "Enteric Microbiota" OR "Gut Flora" OR "Gastrointestinal Flora" OR "Intestinal Flora" OR "Gastrointestinal Microbial Community" OR "GI Microbiota" OR "Enteric Bacteria") OR (Microbiome AND (Gastrointestinal OR Gut OR Intestinal)) OR (Microbiota AND (Gastrointestinal OR Gut OR Intestinal OR Enteric)) OR (Microflora AND (GI OR Gastrointestinal OR Gut OR Intestinal)) | 12037 |
|  | #3 #1 AND #2 | **12** |
